# Supplementary material for: Assembly status transition offers an avenue for activity modulation of a supramolecular enzyme
Source: eLife. 2021 Dec 13;10:e72535. doi: 10.7554/eLife.72535 (PMC8668187; doi:10.7554/eLife.72535)
Supplement: Supplementary file 1. [file elife-72535-supp1.docx]

|  | Plants | | Mammals | | Bacteria | |
| --- | --- | --- | --- | --- | --- | --- |
| Model | Maize  (pdb 2d3a) | Medicago truncatula  (pdb 4is4) | Human  (pdb 2ojw) | Canine  (pdb 2uu7) | Mycobacterium tuberculosis  (pdb 1hto) | Salmonella typhimurium (pdb 1f52) |
| GmGSβ2 (pdb 7v4h) | 0.67 Å  (326) | 0.56 Å  (273) | 0.74 Å  (282) | 0.67 Å  (287) | 3.09 Å  (225) | 3.14 Å  (235) |
| CsGSIb^Dec^  (pdb 7v4i) | 0.77 Å  (337) | 0.96 Å  (308) | 0.88 Å  (301) | 0.83 Å  (297) | 4.17 Å  (257) | 3.12 Å  (239) |

**Supplementary File 1**

**Structural comparisons of GmGSβ2 and CsGSIb^Dec^ with glutamine synthetase from various species.**

Structural comparisons of the glutamine synthetases from different species. At the top is the r.m.s.d value and the number in parentheses at the bottom is the number of residues aligned. The pdb codes used for structural alignments are indicated.
