# Supplementary material for: Assembly status transition offers an avenue for activity modulation of a supramolecular enzyme
Source: eLife. 2021 Dec 13;10:e72535. doi: 10.7554/eLife.72535 (PMC8668187; doi:10.7554/eLife.72535)
Supplement: Supplementary file 2. [file elife-72535-supp2.docx]

**Supplementary File 2**

**Cryo-EM data collection, refinement, and validation statistics.**

|  | CsGSIb^Dec^ (PDB 7V4I) | CsGS1b^Pen^ (I)  (PDB 7V4J) | | | CsGSIb^Pen^ (II)  (PDB 7V4K) | | CsGSIb^Pen^ (III) (PDB 7V4L) | GmGSβ2 (PDB 7V4H) |
| --- | --- | --- | --- | --- | --- | --- | --- | --- |
| Data collection | | | | | | | | |
| Magnification | 29000 x | | 29000 x | 29000 x | | 29000 x | | 29000 x |
| Voltage (kV) | 300 | | 300 | 300 | | 300 | | 300 |
| Electron exposure (e−/Å2 ) | 51 | | 51 | 51 | | 51 | | 51 |
| Defocus range (μm) | -1.6 ~ -2.3 | | -1.6 ~ -2.3 | -1.6 ~ -2.3 | | -1.6 ~ -2.3 | | -1.6 ~ -2.3 |
| Pixel size (Å) | 0.505 | | 0.505 | 0.505 | | 0.505 | | 0.505 |
|  | | | | | | | | |
| Reconstruction and Model composition | | | | | | | | |
| Symmetry imposed | D5 | | C5 | C5 | | C5 | | D5 |
| Chains | 10 | | 5 | 5 | | 5 | | 10 |
| Nonhydrogen atoms | 27680 | | 9850 | 9260 | | 8966 | | 27340 |
| Protein residues | 3560 | | 1275 | 1195 | | 1140 | | 3520 |
|  | | | | | | | | |
| Refinement | | | | | | | | |
| R.m.s. deviations | | | | | | | | |
| Bond lengths (Å) | 0.003 | 0.003 | | | 0.004 | | 0.004 | 0.004 |
| Bond angles (°) | 0.585 | 0.670 | | | 0.660 | | 0.696 | 0.589 |
| Model-to-map fit (CC) | 0.74 | 0.52 | | | 0.64 | | 0.57 | 0.82 |
| Map resolution (Å) | 3.3 | 3.5 | | | 3.6 | | 3.4 | 2.9 |
| FSC threshold | 0.143 | 0.143 | | | 0.143 | | 0.143 | 0.143 |
|  |  |  |  |  |  |  |  |  |
| Validation | | | | | | | | |
| MolProbity score | 1.91 | 2.28 | | | 1.98 | | 2.24 | 1.63 |
| Clashscore | 10.27 | 19.49 | | | 17.27 | | 17.81 | 7.07 |
| Rotamers outliers (%) | 0.00 | 0.00 | | | 0.20 | | 0.00 | 0.00 |
| Ramachandran plot | | | | | | | | |
| Favored (%) | 94.46 | 91.84 | | | 96.33 | | 91.87 | 96.37 |
| Allowed (%) | 5.54 | 8.16 | | | 3.67 | | 8.13 | 3.63 |
| Outliers (%) | 0.00 | 0.00 | | | 0.00 | | 0.00 | 0.00 |
