# Supplementary material for: Assembly status transition offers an avenue for activity modulation of a supramolecular enzyme
Source: eLife. 2021 Dec 13;10:e72535. doi: 10.7554/eLife.72535 (PMC8668187; doi:10.7554/eLife.72535)
Supplement: Transparent reporting form [file elife-72535-transrepform1.docx]

***eLife’s* transparent reporting form**

We encourage authors to provide detailed information *within their submission* to facilitate the interpretation and replication of experiments. Authors can upload supporting documentation to indicate the use of appropriate reporting guidelines for health-related research (see [EQUATOR Network](http://www.equator-network.org/%20)), life science research (see the [BioSharing Information Resource](https://biosharing.org/)), or the [ARRIVE guidelines](http://www.plosbiology.org/article/info:doi/10.1371/journal.pbio.1000412) for reporting work involving animal research. Where applicable, authors should refer to any relevant reporting standards documents in this form.

If you have any questions, please consult our Journal Policies and/or contact us: [editorial@elifesciences.org](mailto:editorial@elifesciences.org).

**Sample-size estimation**

- You should state whether an appropriate sample size was computed when the study was being designed
- You should state the statistical method of sample size computation and any required assumptions
- If no explicit power analysis was used, you should describe how you decided what sample (replicate) size (number) to use

Please outline where this information can be found within the submission (e.g., sections or figure legends), or explain why this information doesn’t apply to your submission:

All tea seedlings of the similar size (at least 30 seedlings) were used for knockdown of Cs14-3-3-1a in tea plant hairy roots (fig 5g,5h,5i,5j).

**Replicates**

- You should report how often each experiment was performed
- You should include a definition of biological versus technical replication
- The data obtained should be provided and sufficient information should be provided to indicate the number of independent biological and/or technical replicates
- If you encountered any outliers, you should describe how these were handled
- Criteria for exclusion/inclusion of data should be clearly stated
- High-throughput sequence data should be uploaded before submission, with a private link for reviewers provided (these are available from both GEO and ArrayExpress)

Please outline where this information can be found within the submission (e.g., sections or figure legends), or explain why this information doesn’t apply to your submission:

All enzymatic assays were performed independently at least three times, and the activity is shown with standard error of the mean (Fig.2b, 2h, S2, S3b and 5j). The information are given in the corresponding Figure Legends. For steady state Michaelis Menten kinetics three replicates were performed for every substrate concentration (minimum of 7), the full curves are shown in figures 2c, 2i, and 2j, and kinetics parameters (with standard error of the mean) are given in the corresponding figures. No replicate was excluded from the data.

At least 5 independent transgenic hairy roots for Cs14-3-3-1a and GUS genes were randomly selected and examined. Knockdown of Cs14-3-3-1a in tea plant hairy roots were performed with five biological replication and three technical replication for each sample to evaluate gene expression levels and amino acid contents. No replicate was excluded from the data analysis.

The transcriptome data were also obtained by three independent repeats and no replicate was excluded from the data.

**Statistical reporting**

- Statistical analysis methods should be described and justified
- Raw data should be presented in figures whenever informative to do so (typically when N per group is less than 10)
- For each experiment, you should identify the statistical tests used, exact values of N, definitions of center, methods of multiple test correction, and dispersion and precision measures (e.g., mean, median, SD, SEM, confidence intervals; and, for the major substantive results, a measure of effect size (e.g., Pearson's r, Cohen's d)
- Report exact p-values wherever possible alongside the summary statistics and 95% confidence intervals. These should be reported for all key questions and not only when the p-value is less than 0.05.

Please outline where this information can be found within the submission (e.g., sections or figure legends), or explain why this information doesn’t apply to your submission:

Raw data for expression patterns of Cs14-3-3-1a and Cs14-3-3-1b genes in various tea plant tissues (Fig. 5b):

Gene_id	AB	YL	ML	OL	ST	RT	FL	FR

Cs14-3-3-1a TEA028539 185.46 145.25	61.01	55.83 146.54 139.64 115.54	51.56

Cs14-3-3-1b TEA007216 336.52 372.14 82.44 100.16 265.54 218.33 338.82 174.84

CsGSII-1b	TEA015580 772.28 445.44 373.37 165.85 511.85 749.45	888.29 597.00

Raw data for induction of Cs14-3-3-1a and Cs14-3-3-1b genes by depletion of NH4+ from culture medium (Fig.5c).

Gene_id	0h	6h	12h	24h	48h	72h	240h

Cs14-3-3-1a TEA028539	180.48	218.94	206.79	208.78	206.85 231.71 224.90

Cs14-3-3-1b TEA007216	143.48	159.09	173.03	190.42	189.27 191.89 144.29

CsGSII-1b	TEA015580	291.99	460.38	437.94	438.67	395.31 311.32 421.68

Raw data for expression of Cs14-3-3-1a and Cs14-3-3-1b genes in tea plant roots fed with 10 mM NH4+ (Fig. 5d)

Gene_id	0h	6h	12h	24h	48h	72h	240h

Cs14-3-3-1a TEA028539	180.48	181.68	187.66	177.54	194.05 184.44	185.50

Cs14-3-3-1b TEA007216	143.48	130.29	146.48	183.00	186.93 200.59	192.27

CsGSII-1b	TEA015580	291.99	313.63	296.18	395.96	365.27 334.99	385.19

qRT-PCR analysis relative expression levels were measured using the 2−ΔCt method.

q-PCR (Fig. 5h):

AVERAGE	AVEDEV	p-value

GUS	0.19497653	0.013334002

14-3-3-1a-KD-1	0.078427417	0.00417873	0.002165

14-3-3-1a-KD-2	0.078623245	0.004126562	0.001858

14-3-3-1a-KD-3	0.047938673	0.000929251	0.000465

The Glutamine content in tea plant samples were analyzed by using an amino acid analyzer (L-8900, Hitachi) according to manufacture instruction.

Glutamine content (Fig. 5i):

AVERAGE	AVEDEV	p-value

GUS	0.109930137	0.009800118

Cs14-3-3-1b-KD-1	0.042805749	0.002029093	0.005144

Cs14-3-3-1b-KD-2	0.056371296	0.000185295	0.010098

Cs14-3-3-1b-KD-3	0.071999095	0.000781139	0.018

(For large datasets, or papers with a very large number of statistical tests, you may upload a single table file with tests, Ns, etc., with reference to sections in the manuscript.)

**Group allocation**

- Indicate how samples were allocated into experimental groups (in the case of clinical studies, please specify allocation to treatment method); if randomization was used, please also state if restricted randomization was applied
- Indicate if masking was used during group allocation, data collection and/or data analysis

Please outline where this information can be found within the submission (e.g., sections or figure legends), or explain why this information doesn’t apply to your submission:

N/A

**Additional data files (“source data”)**

- We encourage you to upload relevant additional data files, such as numerical data that are represented as a graph in a figure, or as a summary table
- Where provided, these should be in the most useful format, and they can be uploaded as “Source data” files linked to a main figure or table
- Include model definition files including the full list of parameters used
- Include code used for data analysis (e.g., R, MatLab)
- Avoid stating that data files are “available upon request”

Please indicate the figures or tables for which source data files have been provided:

N/A
